# Advances in medical adhesives inspired by aquatic organisms’ adhesion

**DOI:** 10.1186/s40824-017-0101-y

**Published:** 2017-10-10

**Authors:** Kyu Ha Park, Keum-Yong Seong, Seung Yun Yang, Sungbaek Seo

**Affiliations:** Department of Biomaterials Science, Life and Industry Convergence Institute, Pusan National University, Miryang, 50463 Republic of Korea

**Keywords:** Medical adhesives, Wet adhesion, Bio-inspiration, Coacervation

## Abstract

In biomedicine, adhesives for hard and soft tissues are crucial for various clinical purposes. However, compared with that under dry conditions, adhesion performance in the presence of water or moisture is dramatically reduced. In this review, representative types of medical adhesives and the challenging aspects of wet adhesion are introduced. The adhesion mechanisms of marine mussels, sandcastle worms, and endoparasitic worms are described, and stemming from the insights gained, designs based on the chemistry of molecules like catechol and on coacervation and mechanical interlocking platforms are introduced in the viewpoint of translating these natural adhesion mechanisms into synthetic approaches.

## Background

The high industrial and biomedical demands for adhesives have led to major progresses in the discovery of their molecular mechanisms as well as the development of the surface science and engineering of adhesive materials. In particular, the advances in polymer science and the usage of lightweight materials have been driven by the aerospace and automobile industries [1]. Whereas, the strict requirements (e.g., biocompatibility, toxicity, and strong adhesive performance) of biomedical adhesives have limited the development of wide-ranging products. For example, the performance of adhesives is dramatically reduced underwater or moisturized conditions. For this reason, researchers have endeavored to improve adhesion efficiency in the presence of water or moisture (termed “wet adhesion”). Moreover, medical adhesives require strong wet adhesion at multifaceted physiological conditions (e.g., pH, salts, and biological molecules).

To overcome these challenges of strong wet adhesion, researchers have been interested in how aquatic organisms survive by attachment/adherence underwater or on wet surfaces. With progresses in understanding the mechanisms and key elements of the natural adhesion observed in aquatic organisms, medical adhesives have been developed via mimicking the adhesion procedures or utilizing the crucial functional groups. The most investigated study is to develop synthetic adhesives inspired by marine mussels [2–6]. They used chemical moieties, e.g., catechols (an analog of the Dopa group of adhesive mussel foot proteins) for tailoring synthetic adhesives. In addition, unique formulation of coacervation (critical step in the formation of the protein-based underwater adhesives) were utilized for constructing efficient wet adhesives.

The aim of this review is to give a brief introduction of various medical adhesives and the challenging aspects of wet adhesion. This review will cover three examples of aquatic organisms’ adhesion – marine mussels, sandcastle worms, and endoparasitic worms, and their insights that can be translated to synthetic platforms and an overview of current synthetic adhesives for biomedical applications.

## Medical adhesives

An effective adhesive requires appropriate materials and adhesion techniques corresponding to diverse biomedical circumstances (host environments), because biological hosts respond differently to the adhesives used for hard or soft tissues.

### Hard tissue adhesives

**Fig. 1:**
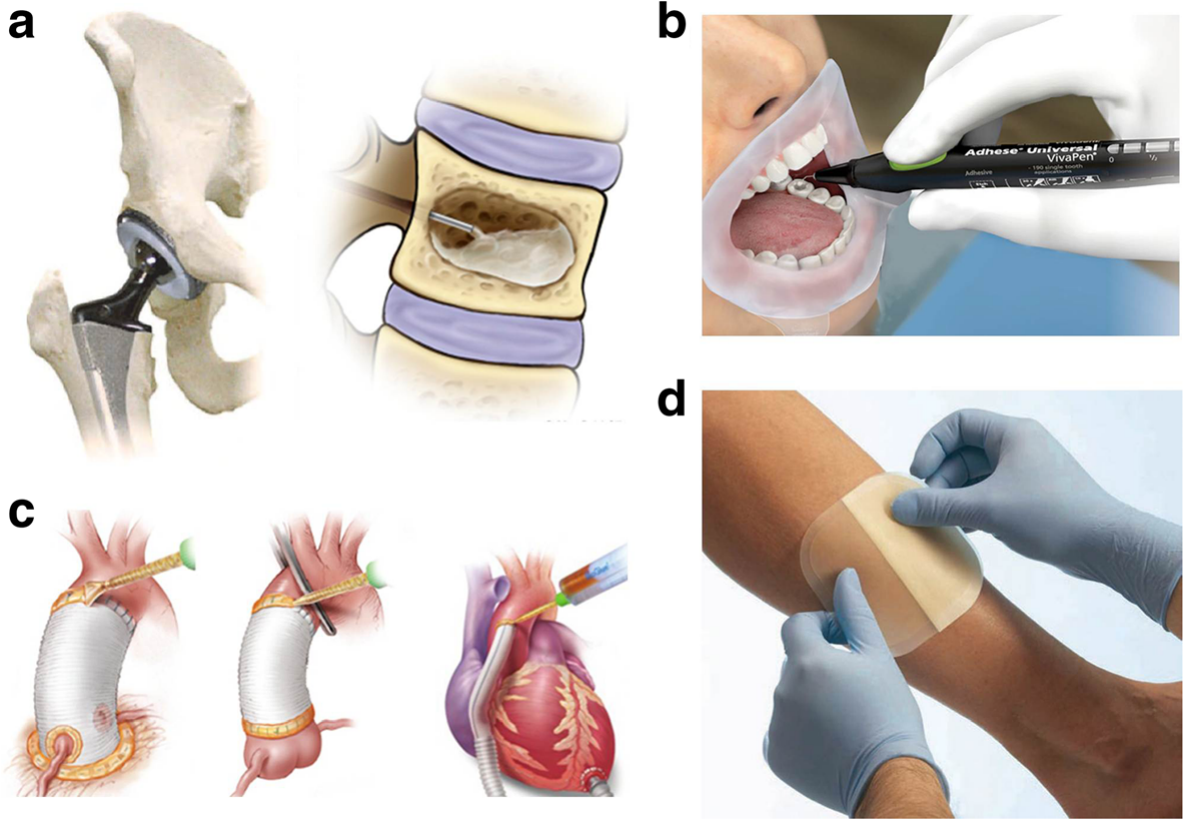
Examples of medical adhesives. **a** Bone cements are used for total hip/joint replacement surgeries and vertebroplasty. Reproduced/edited from Reference [8] **b** Tooth cement/adhesive (Ivoclar Vivadent) for direct and indirect bonding to tooth. **c** BioGlue (Levi BioTECH) is a two-component surgical adhesive/sealant composed of purified bovine serum albumin and glutaraldehyde. **d** Medical patches

Bone and tooth cements are the most used examples of hard tissue adhesives. In joint replacement surgery, the bone cement fills and localizes in the space between the implant and the bone, thereby acting as an implant fixation [7] and transferring the mechanical load from the implant to the bone [8–10] (Fig. 1a, left). Vertebroplasty also uses bone cement to fill, harden, and stabilize a fractured spine bone, by injection through the skin, and prevent further collapse [11, 12] (Fig. 1a, right). Although these are successful applications overall, a sturdier interfacial bond between the implant and bone cement is still required [13].

Poly(methyl methacrylate) (PMMA) has been extensively used for bone cements because the acrylic cement hardens to ~90% of its final mechanical properties within a short time (13–18 min) [8], enabling load bearing and offering immediate stability. However, PMMA-based bone cements have two major limitations. First, PMMA does not have intrinsic adhesive properties, and only acts as a space filler to closely hold the implant against the bone [14]. Such a weak interfacial link between the cement and bone (or implant) results in implant failure [15]. Second, PMMA is a brittle, notch-sensitive material. Although its Young’s modulus (~2 GPa) is 1–2 times higher than that of the surrounding cancellous bone, it is still ~100 times lower than that of the metal prosthesis [16]. Thus, the interspatial bone cement needs to be a shock-buffering spacing between an inflexible bone and a hard implant [14].

Tooth cements (Fig. 1b) have been used for various dental applications, such as a luting agent or for protecting pulps from injury. They help in sealing or fixing and casting the filling substance to both the dentin and enamel. Most of these materials are hard and/or brittle because the load-bearing polymer composites include metallic or ceramic fillers that are hardened by an acid–base reaction [17] or polymerization [18].

Additionally, dental primers have been applied as a way of priming a tooth surface and simultaneously enhancing the adhesion or bonding of the bulk resin composites. For the priming of inorganic fillers, such as silicate minerals, silane-based primers are most commonly used. However, the silane grafting chemistry uses potentially toxic chemicals [19, 20] and tough processing [21, 22] and, therefore, there is a great demand for alternative dental primers.

### Soft tissue adhesives

Soft tissue adhesives are generally planned to be used for transitory or short-term purposes, where they can be removed or degraded when wound healing has progressed sufficiently. For integration of the adhesive with soft tissues that are surrounded by wet tissue fluid or blood, the adhesive needs to be spread easily on the surface and show effective wet adhesion in an adequate working time [23].

The most common examples of soft tissue adhesives are bioglues or sealants [24] (Fig. 1c) and patches [25] (Fig. 1d). Bioglues are usually applied as surgical adhesives in cardiovascular, neurological, and soft tissue surgeries. One such example, BioGlue (Levi BioTECH), was demonstrated to lessen bleeding during cardiac procedures (e.g., aortic dissection, and replacement and implantation of biomedical devices).

In particular, mucosal tissues are required for protection against external and harmful stimuli as well as for treatment with controlled drug delivery. Mucosal adhesives are polymer-based drug delivery platforms, where the degree of cross-linking, the chain length, and the presence of various functional groups in the polymer determine the degree of adhesive bonding and the successful control of drug delivery to the target sites [26].

Patch-type adhesives are also currently used in the clinical field owing to their advantages, including reduced operation times and enhanced tissue handling in a large area. In particular, a glue-coated patch is commonly used as a conventional skin adhesive. However, owing to the allergic reactions and skin irradiation encountered with use of conventional skin adhesives, the fabrication of such types of adhesives without chemical methods is required [27]. Introducing micro- or nanostructures onto the surface of patches has been proven to increase soft tissue adhesion with minimal tissue irritation. Inspired by gecko feet, Geim et al. [28] demonstrated enhanced adhesion using micropatterned poly(imide) films prepared by photolithography and dry etching techniques. The adhesive strength was related to the number of polyimide microstructures present. However, the adhesive performance of the microfabricated patches diminished when submerged in a moist environment, such as bloody tissue or sweaty skin, because of decreased intermolecular interactions. To overcome this limitation, nanofabricated pillar arrays coated with mussel-inspired polymeric glue have been developed [29]. The poly(dimethylsiloxane) nanopillar films coated with poly(dopamine methacrylamide-co-methoxyethyl acrylate) showed reversible adhesion under both dry and wet conditions. Tissue adhesion is highly affected by the chemical and physical properties of the tissue surface. Since tissues have a surface roughness in the range of a few microns to a few millimeters, it is difficult to form a high level of adhesion when the two surfaces of the tissues are not in contact. To achieve universal tissue adhesion regardless of surface conditions, mechanical interlocking-based adhesion is advantageous. Mesh-type adhesion patches with club-shaped hooks have been shown to provide strong adherence to the internal organs of hernias, via entanglement with the tissue surface [30]. In addition, if the hook is made of a biodegradable polymer, it can be easily removed after a certain period of time.

## Wet adhesion

The water in most cells and tissues consist of ~70% by weight as the medium. Additionally, cells, tissues, and implants are typically surrounded by saline water (e.g., blood plasma, lymph, etc.). In the viewpoint of biomedical adhesion, the medium unfortunately creates limited durable binding between the host biological system and the medical adhesive [31], because the water or moisture acts as a surface contaminant or weak boundary layer at the bond interface. This reduced adhesion performance in the presence of water or moisture occurs with most synthetic adhesives. The weakened performance is known to be influenced by complex reasons, such as the hydrolysis of polymers, moisture-induced plasticization, swelling, and erosion [32].

**Fig. 2:**
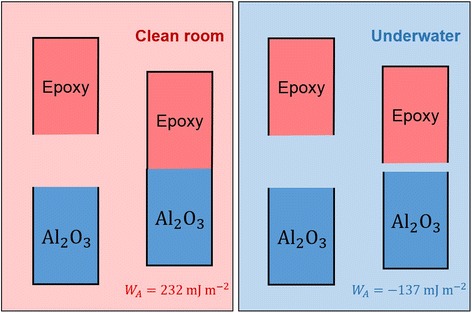
Schematic illustration of the effect of water on adhesion. The work of adhesion (*W*_*A*_) is resulting from the summation of the surface energy products; dispersion interactions (γ_1_^d^ γ_2_^d^) and polar interactions (γ_1_^p^ γ_2_^p^) under clean-room (left) and wet (right) conditions. Reproduced/edited from Reference [31]

The effect of water on the adhesion has been explained, both theoretically and experimentally, as interfacial energies from the summation of electrostatic, polar, and dispersion forces at the adhesion interface [33]. As an example, consider the adhesion between an epoxy adhesive and an aluminum substrate under clean-room and wet conditions, in terms of interfacial energies [21] (Fig. 2). Herein, the work of adhesion (*W*_*A*_ = 2γ_i_, where γ_i_ is the interfacial energy) can be determined by the polar (γ^p^) and dispersive (γ^d^) components of each interfacial energy. Under vacuum, the work of adhesion is positive (e.g., *W*_*A*_ = 232 mJ·m^−2^) and quite strong, whereas in water, the work of adhesion is negative (e.g., *W*_*A*_ = −137 mJ·m^−2^) and is not noticeably effective. This clearly shows the challenge of ensuring good bonding between conventional epoxy adhesives and metal substrates, and why a strategy to overcome the limitation of wet adhesion needs to be designed for medical adhesives. In the evolution of natural adhesion, the water medium has contributed a decisive role. The following sections review the adhesion mechanisms of aquatic organisms and synthetic options gained from the insights of the natural adhesion, and design principles for successful biomedical underwater adhesives.

## Adhesion mechanisms of aquatic organisms and their inspired medical adhesives developments

For aquatic organisms, attachment (or adherence) is a survival strategy in tough water environments [34, 35]. For example, marine mussels/giant clams and barnacles adhere to rock surfaces by using their byssus and secreted cement proteins, respectively [34, 36]. Aquatic larvae and black fly pupae anchor to environmental surfaces using adhesive proteins [37]. Here, unique motifs (viz., the coacervate formation/platform, and mechanical interlocking mechanism) will be discussed with respect to their role in the natural adhesion.

### Marine mussels

**Fig. 3:**
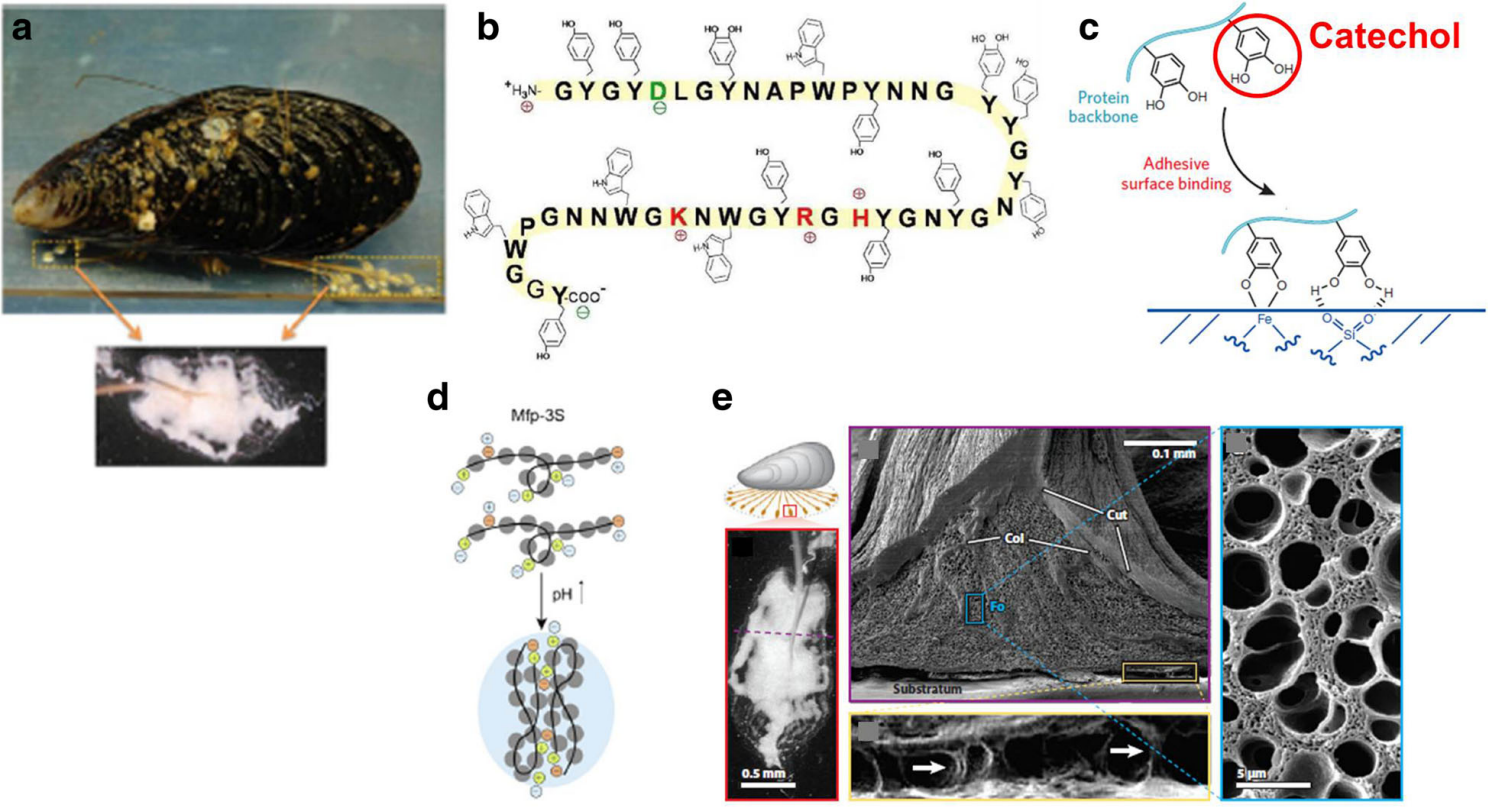
Adhesion mechanisms of marine mussel foot proteins (Mfps). **a** A representative sequence of Mfp-3 from mussel plaque. **b** The amino acid sequence of Mfp-3. **c** Schematic illustration of mussel adhesion with the catechol moiety. **d** A scheme of one (or self-) complex coacervate. **e** Ultrastructure of a byssal adhesive plaque. The figure a-e was reproduced/edited from References [31, 38, 39], respectively

Marine mussels [38] (Fig. 3a) attach to hard surfaces (e.g., minerals and metals) in the intertidal zone, where waves with and without suspended sand often exceed 25 m·sec^−1^ velocities. One of the intriguing features of wet adhesion in marine mussels and sandcastle worms is the metastable water-insoluble fluids that resist, or are separately dispersed in, the surrounding seawater [38]. In mussels, these adhesive fluids consist of the Mfps as highly concentrated, intrinsically unstructured polyelectrolytes [38] (Fig. 3d) that solidify rapidly upon equilibration with seawater. These interfacial Mfps have an unusually high abundance (28–34 mol%) of aromatic residues, including tyrosine (Y), tryptophan (W), and Dopa (a posttranslationally modified form of tyrosine, Y′) [38] (Fig. 3b). Among these, Dopa is now accepted as one of the key functional groups for wet adhesion owing to its strong bidentate binding to oxide mineral surfaces [39] (Fig. 3c), and has been incorporated into synthetic polymers to mimic wet bioadhesion [40–44].

The interface between a marine mussel’s byssal adhesive plaque and a glass substrate resembles a porous-like structure but with pillar-shaped attachment [31] (Fig. 3e). Such structure and shape at the interface can be considered promising architectures for the design of underwater adhesives.

**Fig. 4:**
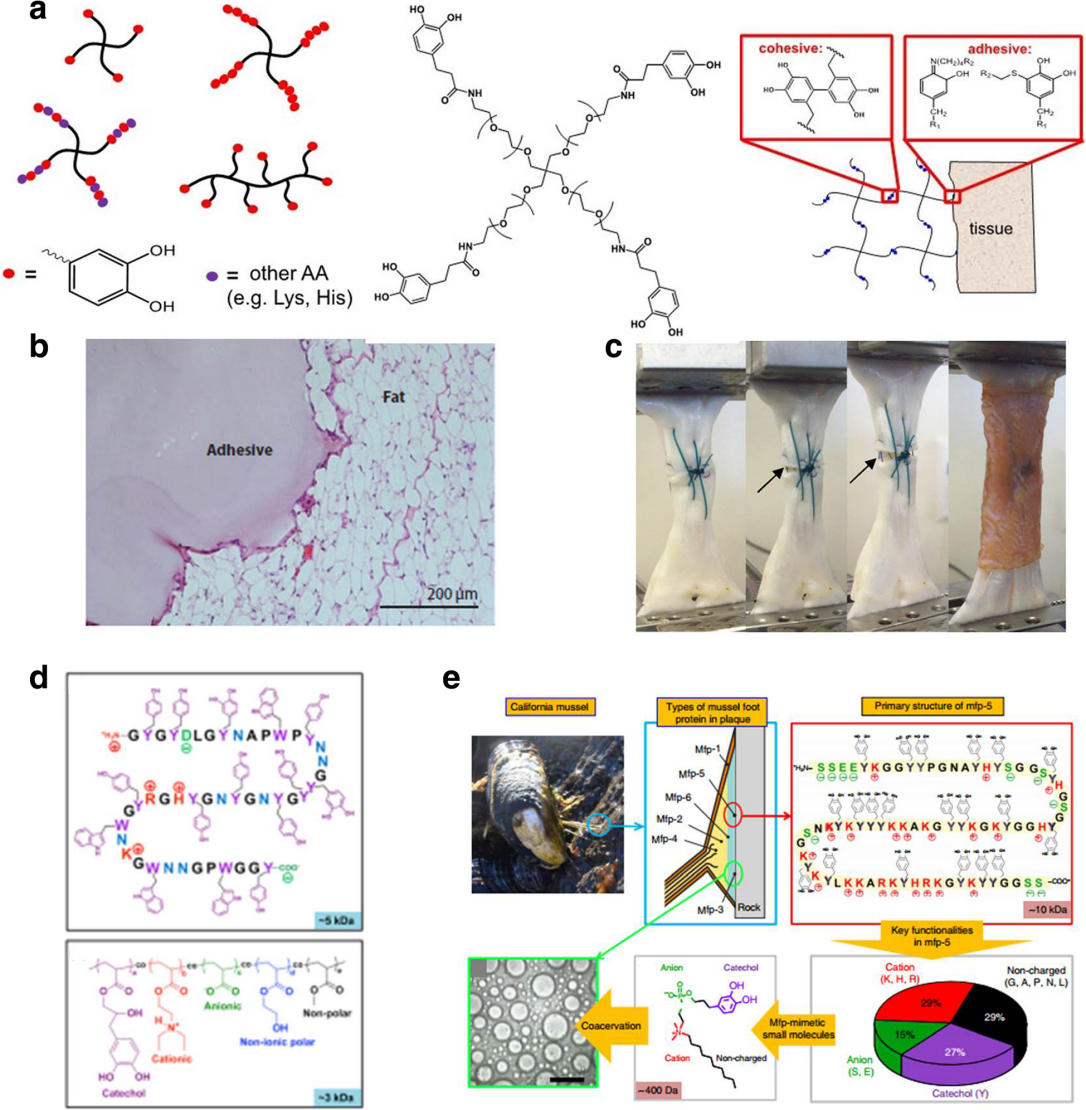
Synthetic approaches inspired by mussel adhesion. **a** Schematic illustration of mussel adhesive protein-inspired mimetic polymer systems. Dopa or a catechol mimic of Dopa covalently coupled to polymer chain ends or as side chains of polymerizable catechol monomers. **b** Histological results from implantation of the PEG-catechol hydrogel into mice. **c** Images of tissue adhesives with adhesive properties that combine high strength. **d** Key features of mussel foot protein and its synthetic homolog. **e** Key features of natural and translated mussel adhesion. The figure b, d, e was reproduced/edited from References [31, 52, 54], respectively

The chemical functionalization of catechols (an analog of the Dopa group of adhesive Mfps) into synthetic polymers is the most common way to construct mussel-inspired adhesives [45] (Fig. 4a). Owing to this straightforward and economical method of constructing synthetic molecules, this strategy has overwhelmed the mussel-inspired adhesive community for 10–20 years. Like the native mussel proteins, the catechol in these polymers contribute to interfacial adhesion and cross-linking.

As an example of such catechol-functionalized polymers, polyethylene glycol (PEG)-catechol adhesives have been studied in biomedical applications, where the adhesion performance and interfacial progress (i.e., tissue biocompatibility, and integrity of the tissue and the adhesive) were investigated in mice [46]. After implantation of the polymeric adhesives, no noticeable inflammatory cell infiltrates and fibrotic capsule formation appeared at the given time [31] (Fig. 4b, left). After several months, vascularization was well structured on the implant site of the catechol polymer-immobilized islets [31] (Fig. 4b, right). Thus, the PEG-catechol adhesives demonstrated biocompatibility in biomedical applications and appropriate integrity toward the host tissue. Likewise, Lee’s group focused on developing tissue adhesives with tunable physical, mechanical, and adhesive properties that combined high strength with the ability to support tissue ingrowth and wound healing [47–51] (Fig. 4c). Additionally, the Waite and Kollbe group considered other constitutional features of interfacial Mfps, such as cationic residues (lysine, K), anionic residues (aspartic acid, D), nonionic polar residues (asparagine, N), and nonpolar residues (alanine, A), to create mussel-inspired synthetic wet-adhesion systems [19, 52–54] (Fig. 4d and e).

### Sandcastle worms

**Fig. 5:**
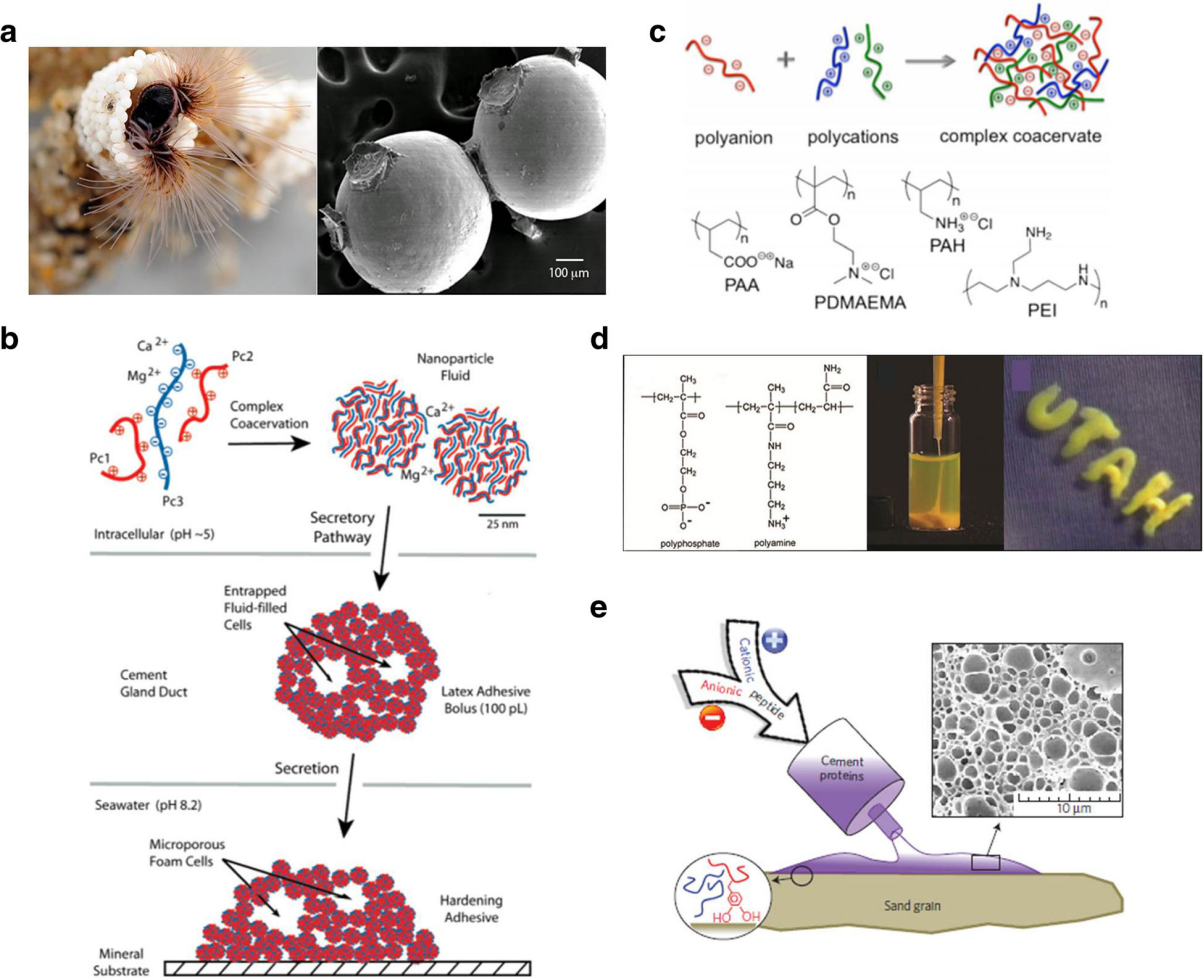
Synthetic approaches inspired by sandcastle worm adhesion insights. **a** Images of sandcastle worms and sandcastle glue. **b** Adhesion model. Within secretory cells of the cement glands, a mixture of the oppositely charged adhesive proteins and divalent cations condense into a nanoparticulate fluid phase through complex coacervation. **c** Schematic illustration of ternary complex coacervate formation. **d, e** Sand castle worm-inspired synthetic molecules and coacervation. The figure **b, c, e** was reproduced/edited from References [55, 60, 63], respectively

In sandcastle worm cement [55] (Fig. 5a), in the presence of both polyanions (polyphosphoserine-rich proteins) and polycations (lysine-rich proteins), fluid–fluid phase separation is modeled as a complex coacervation process, leading to a polyelectrolyte-depleted equilibrium [40, 49, 56] (Fig. 5b). Complex coacervation results from neutralization of the oppositely charged polyelectrolytes, coupled with the concomitant release of the counterions [55] (Fig. 5c), and confers unusual properties to the coacervate phase, including relatively high diffusion coefficients of the solute and solvent molecules, high concentrations, relatively low viscosity, and low interfacial energy, which are all highly favorable to dispensing adhesion under water [57–59].

One good example of such adhesive platforms using complex coacervation is the synthetic polyelectrolytes established by the Stewart group [57, 60], which mimic the polyelectrolytic proteins in the sandcastle glue [61] (Fig. 5d). Those authors were inspired by the dense, phase-separated fluid of the sandcastle glue-like polyelectrolytic proteins. They formed various supramolecular platforms—from colloidal structures to insoluble precipitates or ionic gels—and optimized them into sandcastle glue-mimicking coacervates by controlling the solution conditions and polymer structures. For condensation of the polyelectrolytes, an entropic driving force was employed; such as electrostatic charge neutralization between the polymeric charges to displace small counterions and water.

The Waite group also developed concrete underwater constructs inspired by the sandcastle worm’s glue-like protein mortar [62]. This worm uses a significant principle for the design of such structures by selecting sand granular particles with a protein mortar glues [63] (Fig. 5e, left). Upon deposition onto the particle surfaces, the coacervate becomes three-dimensional porous solid structure regarded as by incorporation of the coacervates and structural maturation of the metal ion- protein complexes. Based on cross-links by oxidized l-Dopa, the tubular walls were then cured [39, 63, 64] (Fig. 5e, right).

### Endoparasitic worms

**Fig. 6:**
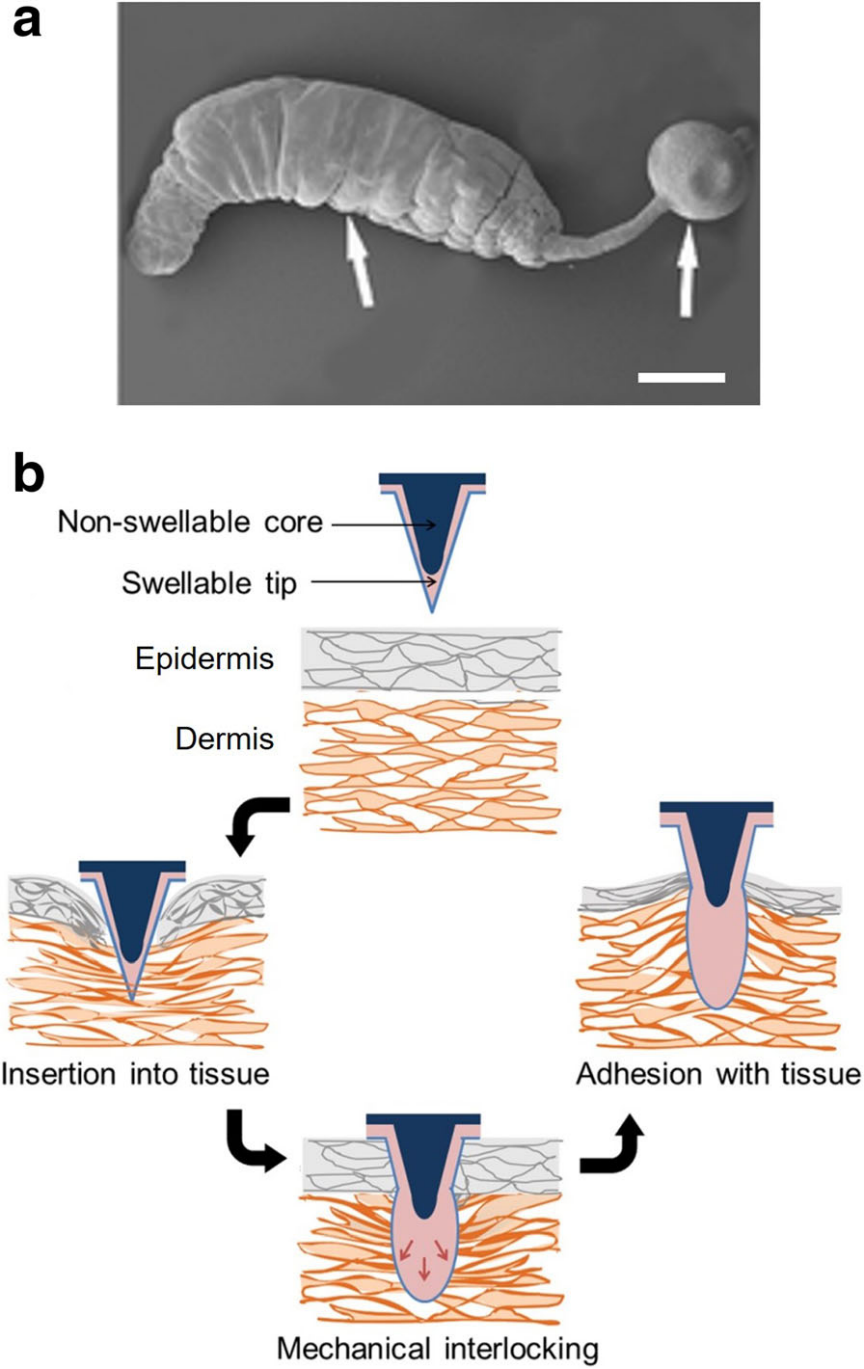
Mechanical interlocking-based adhesion system inspired by endoparasitic worms. **a** Photograph of the inflated proboscis of *Pomphorhynchus laevis* (Scale bar: 100 μm). Reproduced/edited from Reference [68] with permission. **b** Illustration showing mechanical interlocking of the double-layered microneedle with a water-responsive tip following its penetration into skin tissue

These worms are organisms that live inside the body of host animals in their developmental or adult stages. Several internal parasites have evolved to adhere to the intestinal wall of their host by using specialized parts, such as hooks or suckers [65]. During attachment, they feed on the ingesta in the host intestine or suck blood or epithelial cells from the mucosal layer within the intestine. *Pomphorhynchus laevis*, known as the spiny-head worm, uses an inflatable proboscis to secure a parasitic position following penetration of the host intestine wall (Fig. 6a) [66, 67]. This mechanical interlocking-based attachment provides strong adhesion onto the fish intestinal wall. Inspired by such endoparasitic worms, a patch-type microneedle adhesive enabling mechanical interlocking with soft tissues has been recently developed to achieve wet tissue adhesion [68]. Yang et al. [68] prepared a double-layered microneedle consisting of swellable tips and a non-swellable core, providing firm tissue adhesion based on mechanical interlocking following its insertion into the tissue (Fig. 6b). The microneedle adhesive showed good attachment to multiple wet tissues, such as skin, muscle, and intestine, in a minimally invasive manner. Since drugs can be loaded into the swellable tips of the microneedle adhesive, sustained release of the loaded drug to the mechanically interlocked target tissue was achieved through the swollen tips [69].

## Conclusions

In the development of medical adhesives, wet adhesion is an inherent and considerable point of challenge. Through bioinspired or biomimetic ways of translating natural adhesion mechanisms into synthetic approaches, it is possible to save the time-consuming synthesis of adhesives. As reviewed herein, translation of the natural adhesion mechanisms of the marine mussel, sandcastle worm, and endoparasitic worms into synthetic platforms—ranging from synthetic molecules and colloidal systems to coacervation and mechanical interlocking processes—will help to realize the design and fabrication of effective underwater adhesives for biomedical applications.
